# Physical activity and adolescent anxiety trajectories: a longitudinal analysis of exercise self-efficacy and body image

**DOI:** 10.3389/fpubh.2026.1825516

**Published:** 2026-05-19

**Authors:** Wei Xiong, Jia-Le Yu, Zi-Quan Zhang, Yun-Xiang Fan

**Affiliations:** 1 College of Physical Education, Changsha University, Changsha, China; 2 College of Physical Education, Nanchang Normal University, Nanchang, China; 3 College of Physical Education, Hunan Normal University, Changsha, China

**Keywords:** developmental pathways, internalizing symptoms, latent class modeling, mental health promotion, psychosocial stability

## Abstract

**Purpose:**

Adolescent anxiety develops heterogeneously, yet most evidence on physical activity remains cross-sectional and variable-centered. This study examined whether baseline physical activity was associated with differential anxiety trajectory membership across one academic year and whether exercise self-efficacy and body image were related to these associations.

**Methods:**

A three-wave longitudinal study was conducted among 1,395 middle school students. Of these, 1,243 provided complete data across all waves. Participants were first stratified into stability-dominant and variability-dominant subgroups based on cross-wave similarity. Anxiety trajectories were then estimated within the variability-dominant subgroup using latent class trajectory modeling. Associations of baseline physical activity with later exercise self-efficacy, body image, and anxiety trajectory membership were examined using linear regression, multinomial logistic regression, and bootstrap-based indirect pathway models controlling for grade, sex, and age.

**Results:**

Most adolescents with complete data were classified into the stability-dominant subgroup (76.7%), whereas 23.3% were classified into the variability-dominant subgroup. Within the variability-dominant subgroup, a 3-class solution was retained, comprising Stable Elevated (60.0%), Declining Improvement (18.6%), and Increasing Risk (21.4%) trajectories. Baseline physical activity differentiated subsequent trajectory membership and positively predicted later exercise self-efficacy and body image. Exercise self-efficacy also positively predicted later body image. However, the hypothesized serial indirect pathway through exercise self-efficacy and body image was not consistently supported across trajectory contrasts.

**Conclusion:**

Physical activity was associated with differential anxiety trajectory membership, but these associations unfolded within a broader pattern of psychosocial stability. Exercise self-efficacy showed a more consistent role than body image, whereas the hypothesized serial pathway was not robustly supported. Physical activity may therefore be more relevant to developmental positioning across anxiety trajectories than to a simple uniform symptom-reduction model.

## 1 Introduction

Adolescence is a developmental period marked by heightened vulnerability to anxiety disorders and anxiety-related symptomatology. Epidemiological evidence consistently shows that the incidence and exacerbation of anxiety increase during early and middle adolescence, with substantial heterogeneity in onset, severity, and persistence (1, 2). Elevated anxiety during this period predicts later depressive disorders, substance misuse, academic impairment, and long-term psychosocial dysfunction (3, 4). Importantly, anxiety does not follow a single uniform course. Longitudinal research using person-centered approaches, such as latent class and growth mixture modeling, has identified distinct developmental patterns rather than a single average trajectory (5). These findings challenge static views of adolescent anxiety and highlight the need to examine factors associated with differential trajectory membership rather than cross-sectional symptom levels alone.

Despite clear evidence of developmental heterogeneity, much of the literature on protective factors for adolescent anxiety remains variable-centered and cross-sectional. Physical activity, in particular, has been consistently associated with lower anxiety symptoms in youth populations (6). Moderate-to-vigorous activity has been linked to reductions in generalized anxiety, social anxiety, and negative affect (7). Yet most studies assess anxiety at a single time point or estimate average symptom change across a sample, leaving it unclear whether physical activity is associated with differential developmental pathways, such as lower likelihood of persistent elevation, decreasing risk over time, or reduced probability of trajectory escalation. Addressing this gap requires integrating exercise research with longitudinal trajectory-based models of emotional development.

Beyond whether physical activity is associated with anxiety trajectories lies a deeper theoretical question: through what psychological processes might such associations emerge? Emerging evidence suggests that links between physical activity and anxiety are unlikely to be purely direct and may instead involve intermediary self-regulatory and self-evaluative processes (8). Social cognitive theory proposes that repeated mastery experiences strengthen self-efficacy beliefs, which in turn shape cognitive appraisal and emotional responding (9). Exercise self-efficacy, defined as confidence in one’s ability to initiate and sustain physical activity, has been associated with lower anxiety and more adaptive emotional functioning in adolescents (10, 11). Adolescents with stronger exercise-related self-efficacy may perceive challenges as more manageable, experience greater control, and show lower vulnerability to anxiety maintenance.

Adolescence is also marked by heightened salience of physical appearance and increased sensitivity to peer evaluation. Body image dissatisfaction is highly prevalent during this period and has been linked to both concurrent and prospective anxiety symptoms, particularly in the domains of social anxiety and evaluative threat (12, 13). Sociocultural models propose that internalization of appearance ideals and negative self-evaluation amplify susceptibility to social threat and anxiety-related distress (14). Physical activity may be associated with more positive body-related perceptions through improvements in physical competence, bodily awareness, and embodied self-evaluation. At the same time, self-efficacy and body image may not operate as fully independent processes. A theoretically plausible possibility is that exercise strengthens self-efficacy, which may then support more adaptive body-related evaluations. This perspective motivates testing a temporally ordered indirect pathway rather than assuming that each mechanism acts in isolation.

Although prior studies have examined self-efficacy or body image as potential mediators separately (11, 13), relatively few investigations have tested these constructs within a longitudinal, person-centered framework that differentiates persistent, improving, and escalating anxiety patterns. Consequently, it remains unclear whether physical activity is associated with differential developmental trajectories of anxiety, and whether exercise self-efficacy and body image contribute to these associations through temporally ordered indirect pathways.

The present study addressed these questions using a longitudinal framework designed to capture heterogeneity in adolescent anxiety across one academic year. By moving beyond cross-sectional associations, it examined whether physical activity was associated with anxiety trajectory membership and whether exercise self-efficacy and body image were related to those associations. Therefore, the study sought to clarify how physical activity may be linked to heterogeneous anxiety development and to inform the integration of physical education and mental health promotion in school settings.

## 2 Literature background and hypothesis development

### 2.1 Anxiety as a heterogeneous developmental process

Contemporary developmental psychopathology no longer treats adolescent anxiety as a single, uniform condition. Rather, anxiety is increasingly understood as a dynamic process that unfolds through ongoing interactions between personal vulnerabilities and environmental demands across time (15). Within this framework, adolescents may begin from different levels of risk and follow different developmental courses depending on how emotional, cognitive, and social processes accumulate or adapt over time.

Person-centered longitudinal research supports this view. Instead of identifying only average trends at the sample level, studies using latent class or growth mixture approaches have shown that adolescents often separate into distinct anxiety pathways, such as persistently elevated, escalating, declining, or relatively resilient trajectories (16, 17). These developmental distinctions are substantively important because trajectory membership is linked to later psychiatric comorbidity, academic difficulties, and interpersonal impairment (18, 19). The key implication is that the study of protective factors should move beyond average symptom reduction and ask whether such factors are associated with the probability of following one developmental pathway rather than another.

This point is especially relevant to physical activity research. Although a large literature links physical activity to lower anxiety, much of that work remains variable-centered and focuses on concurrent symptom levels or mean change across a sample (8). Such designs are valuable, but they cannot determine whether physical activity is associated with the likelihood of belonging to a persistently elevated, improving, or increasing-risk trajectory. If anxiety development is heterogeneous, then the role of physical activity should also be examined at the level of developmental subgroup membership rather than only mean symptom variation.

From this perspective, physical activity may be understood not simply as a behavior correlated with better mood, but as a developmental resource that helps steer adolescents toward more adaptive emotional pathways over time. Repeated engagement in physical activity may shape appraisal processes, self-regulatory capacity, and social experience in ways that reduce the likelihood of remaining on, or shifting toward, a higher-risk anxiety course.

Hypothesis 1: Higher levels of physical activity may be associated with increased likelihood of membership in lower-risk anxiety developmental trajectories.

### 2.2 Exercise self-efficacy as a cognitive-regulatory pathway

The psychological benefits of physical activity are unlikely to be explained solely by physiological activation or energy expenditure. Social cognitive theory instead emphasizes that behavioral experiences influence emotional functioning through cognitive appraisals, especially self-efficacy beliefs (9). Self-efficacy refers to perceived capability to organize and execute actions required to manage prospective situations. Individuals with stronger efficacy beliefs generally appraise demands as more manageable, persist longer under challenge, and show more adaptive emotional responses to stress (20).

In the context of physical activity, exercise self-efficacy reflects adolescents’ confidence in their ability to initiate, maintain, and regulate exercise despite barriers. This confidence is theoretically relevant to anxiety because it bears directly on perceived control, coping expectations, and responses to challenge. Adolescents who repeatedly succeed in physical activity contexts may accumulate mastery experiences that strengthen their sense of behavioral competence. In turn, a stronger sense of competence may reduce helplessness, attenuate exaggerated threat appraisal, and support more adaptive emotion regulation. Empirical work has accordingly linked higher exercise self-efficacy to more favorable emotional functioning and lower anxiety-related symptoms in adolescent samples (21, 22).

Importantly, the present study treats exercise self-efficacy not as a broad trait-like self-evaluation, but as a domain-specific cognitive-regulatory belief grounded in perceived capability for action. This distinguishes it conceptually from body image. Exercise self-efficacy concerns what adolescents believe they can do in the physical domain, whereas body image concerns how they evaluate and feel about their bodies and appearance. The two constructs may be related, but they are not interchangeable: one is fundamentally competence-based and action-oriented, the other evaluative and affect-laden. This distinction is theoretically important because physical activity may first alter perceived capability before influencing broader body-related self-evaluations.

Accordingly, exercise self-efficacy is a plausible pathway through which physical activity becomes associated with anxiety development. If physical activity strengthens adolescents’ confidence in handling challenge, that cognitive shift may lower the probability of following a persistently elevated or worsening anxiety trajectory.

Hypothesis 2: Exercise self-efficacy may partly account for the association between physical activity and anxiety trajectory membership.

### 2.3 Body image as an affective-evaluative pathway

Adolescence is also a period of intensified body awareness and heightened sensitivity to social evaluation. Physical appearance becomes increasingly salient in peer contexts, and body-related judgments often acquire strong emotional significance. Within this developmental context, body image has emerged as an important correlate of adolescent mental health. Body image dissatisfaction has been consistently associated with anxiety symptoms, particularly those involving social evaluation, self-consciousness, and panic-related distress (23, 24).

Sociocultural models help explain this link by proposing that internalized appearance standards and appearance-based comparison heighten vulnerability to evaluative threat (25, 26). Adolescents who view their bodies negatively may become more vigilant to peer judgment, more self-focused in social situations, and more prone to fear of negative evaluation. These processes are central to social anxiety and may also contribute more broadly to anxiety vulnerability (27). Conversely, a more positive body image is associated with greater self-acceptance, lower evaluative concern, and reduced anxiety symptomatology (28).

Physical activity may be relevant to body image through several channels. Engagement in exercise can increase bodily awareness, perceived strength, and feelings of competence, while also offering a context in which the body is experienced functionally rather than judged purely in appearance terms. Such experiences may foster more positive body-related perceptions. However, the association is unlikely to be purely cosmetic. From a developmental standpoint, body image may improve not only because activity changes physical sensations or appearance-related interpretations, but also because repeated successful engagement in physical activity alters how adolescents relate to their bodies more generally.

If negative body image amplifies sensitivity to social threat and anxiety maintenance, then more positive body image may be associated with lower likelihood of persistent or escalating anxiety trajectories.

Hypothesis 3: Body image may partly account for the association between physical activity and anxiety trajectory membership.

Hypothesis 4: Exercise self-efficacy may be positively associated with subsequent body image.

### 2.4 A sequential pathway linking physical activity to anxiety development

Although exercise self-efficacy and body image can each be considered separately, a stronger theoretical account is that they may be developmentally connected. Social cognitive theory suggests that mastery experiences generated through repeated behavioral engagement first shape efficacy beliefs (9). Sociocultural and self-evaluative perspectives suggest that body-related perceptions are then influenced by how adolescents interpret their competence, adequacy, and value in socially visible domains (14, 26). Integrating these traditions suggests that the effects of physical activity may unfold through an ordered process rather than through isolated parallel mechanisms.

More specifically, physical activity may first strengthen exercise self-efficacy by providing repeated experiences of success, persistence, and control. Stronger exercise self-efficacy may then support more adaptive body evaluations, because adolescents who feel capable in the physical domain may be more likely to interpret their bodies in terms of function, strength, and adequacy rather than deficiency or appearance-based threat. Improved body image may in turn reduce self-consciousness and evaluative vulnerability, thereby lowering the likelihood of more adverse anxiety trajectories.

This sequential account offers a more integrated developmental model than treating self-efficacy and body image as unrelated mediators. It also fits the temporal logic of adolescent development, in which behavioral experience may first recalibrate competence beliefs and only subsequently reshape broader self-evaluative and affective processes. Prior studies have often examined self-efficacy or body image in isolation (11, 13), but fewer have tested whether these constructs operate in sequence within a person-centered longitudinal framework that distinguishes persistent, improving, and escalating anxiety pathways.

Accordingly, the present study proposes that physical activity is associated with anxiety trajectory membership not only directly, but also through an ordered pathway linking behavioral mastery, competence belief, and body-related self-evaluation.

Hypothesis 5: Physical activity may show a serial indirect association with anxiety trajectory membership through exercise self-efficacy and body image.

## 3 Methods

### 3.1 Study design

This longitudinal cohort study examined developmental changes in anxiety across one academic year among middle school students in Hunan Province, China. Data were collected at three time points over a 12-month period to enable identification of heterogeneous anxiety trajectories and examination of associated psychological mechanisms.

The study protocol was approved by the Ethics Committee of Hunan Normal University. All procedures complied with the Declaration of Helsinki. Written informed consent was obtained from all participating students and their legal guardians prior to data collection.

### 3.2 Participants

Participants were recruited using stratified cluster random sampling. Four public middle schools were selected from a county in Hunan Province, a region considered educationally and demographically representative within the province. Schools were first stratified by urban and rural location, and two schools were randomly selected from each stratum.

Because the study involved longitudinal follow-up, Grade 9 students were excluded due to anticipated graduation during the study period. Within each selected school, four intact classes were randomly sampled from Grade 7 and four from Grade 8. All students in the selected classes were eligible to participate.

Data were collected at three time points across one academic year: baseline (T1, September 2024), follow-up 1 (T2, March 2025), and follow-up 2 (T3, September 2025). This three-wave design enabled examination of cross-wave stability and longitudinal subgrouping.

### 3.3 Procedures

All three assessments followed identical procedures. Questionnaires were administered in classroom settings by trained research personnel during regular school hours. Completed surveys were collected immediately to ensure data integrity.

To enable longitudinal matching while preserving anonymity, students reported their class and the last four digits of their student identification number. Data were screened for completeness and response consistency following each wave of collection.

### 3.4 Measures

Physical activity was assessed using the Physical Activity Rating Scale (PARS-3) (29). The instrument evaluates exercise intensity, duration, and frequency using three items rated on five-point scales. Intensity and frequency are scored from 1 to 5, and duration from 0 to 4. The total physical activity score is calculated as intensity × duration × frequency. Scores ≤19 indicate low activity, 20–42 moderate activity, and ≥43 high activity. For analysis, physical activity was entered as a three-level categorical variable (low, moderate, high). The reported 21-day test–retest reliability is 0.601 (*p* < 0.01).

Exercise self-efficacy was measured using the Chinese version of the Exercise Self-Efficacy Scale (30). The scale includes nine items rated on a 10-point Likert scale ranging from 0 (“no confidence at all”) to 10 (“very confident”). Higher scores reflect stronger perceived exercise self-efficacy. Cronbach’s alpha coefficients in the present study were 0.890 at T1, 0.891 at T2, and 0.892 at T3.

Body image was assessed using the Body Image States Scale (31). The instrument contains six items rated on a nine-point Likert scale ranging from 1 (“extremely dissatisfied”) to 9 (“extremely satisfied”). Higher scores indicate greater body satisfaction. Cronbach’s alpha coefficients were 0.869 at T1, 0.867 at T2, and 0.870 at T3.

Anxiety symptoms were assessed using the Chinese version of the Self-Rating Anxiety Scale (32). The scale includes 20 items, five of which are reverse-scored. Responses are rated on a four-point Likert scale ranging from 1 (“none or a little of the time”) to 4 (“most or all of the time”). Total scores were treated as continuous indicators of anxiety severity, with higher scores reflecting greater anxiety. Cronbach’s alpha coefficients were 0.850 at T1, 0.853 at T2, and 0.858 at T3.

### 3.5 Statistical analysis

Wave-specific scores were first derived for all study variables. Physical activity was calculated from the PARS-3 as the product of exercise intensity, duration, and frequency, and was categorized as low, moderate, and high. Exercise self-efficacy, body image, and anxiety were represented by wave-specific mean scores. Baseline descriptive statistics were computed for the baseline analytic sample (*n* = 1,395), whereas longitudinal subgrouping, trajectory modeling, multinomial regression, and indirect pathway analyses were conducted using complete-case data from participants with complete three-wave data (*n* = 1,243); participants with incomplete follow-up data were excluded from these analyses. Baseline differences between subgroups were evaluated using independent-samples *t* tests with standardized mean differences for continuous variables and chi-square tests with Cramér’s V for categorical variables. Partial correlations among physical activity, exercise self-efficacy, body image, and anxiety across the three waves were estimated while controlling for grade, sex, and age.

Because the primary objective was to identify heterogeneous developmental trajectories, a preliminary similarity analysis was performed before trajectory modeling. This step was used to distinguish adolescents whose repeated measurements showed very high cross-wave similarity from those who retained sufficient longitudinal variation for trajectory-based analysis. For each construct, similarity across the three waves was evaluated using the three pairwise comparisons (T1–T2, T1–T3, and T2–T3). Given that only three pairwise comparisons were available, the predefined 90% repeat threshold required exact equality across all three comparisons. Participants meeting this high-similarity criterion on at least two of the four constructs were classified into the stability-dominant subgroup, whereas the remainder were classified into the variability-dominant subgroup. Subsequent trajectory and pathway analyses were conducted in the variability-dominant subgroup.

Within the variability-dominant subgroup, anxiety developmental trajectories were estimated using latent class trajectory modeling. Models specifying one to five classes were compared. Model selection was guided by Akaike Information Criterion, Bayesian Information Criterion, entropy, mean posterior probability, minimum class proportion, and substantive interpretability. Solutions yielding very small classes were not retained, even when a single information criterion suggested marginal improvement. After the optimal solution was identified, participants were assigned to trajectory classes according to posterior classification, and classes were labeled on the basis of their temporal patterns.

To examine associations between baseline physical activity and subsequent psychological variables, linear regression models were estimated. Exercise self-efficacy at T2 was regressed on baseline physical activity and covariates, and body image at T3 was regressed on baseline physical activity, exercise self-efficacy at T2, and covariates. To examine associations with trajectory membership, multinomial logistic regression was then used with the stable elevated trajectory as the reference class. Low physical activity served as the reference category for activity-level comparisons. Grade, sex, and age were included as covariates in all models. Regression coefficients, odds ratios, 95% confidence intervals, and two-sided *p* values were reported as appropriate.

Prospective indirect associations with trajectory membership were examined using contrast-specific binary models. In these analyses, exercise self-efficacy at T2 was modeled as a function of baseline physical activity, prior exercise self-efficacy at T1, and covariates; body image at T3 was modeled as a function of baseline physical activity, exercise self-efficacy at T2, prior body image at T1, and covariates; and trajectory-contrast membership was modeled as a function of baseline physical activity, exercise self-efficacy at T2, body image at T3, and covariates. Indirect effects were decomposed into pathways via exercise self-efficacy, via body image, through the serial pathway linking exercise self-efficacy to body image, and as total indirect effects. These estimates were obtained using 5,000 bootstrap resamples and were reported on the model coefficient scale rather than the odds-ratio scale. Given the contrast-specific modeling strategy and the fact that trajectory membership was derived from anxiety measured across T1–T3, these analyses were interpreted as prospective indirect associations rather than strong causal mediation effects. Multicollinearity was evaluated using variance inflation factors.

All analyses were conducted in R packages, including lcmm (version 2.2.2) for latent class trajectory modeling (33) and nnet (version 7.3–20) for multinomial logistic regression. Statistical significance was defined as *p* < 0.05. For bootstrap-based indirect effects, statistical support was inferred when the 95% confidence interval excluded zero.

## 4 Results

### 4.1 Baseline characteristics and longitudinal stability stratification

Of the 1,439 questionnaires collected at baseline, 1,395 met the validity criteria and were included in the baseline analytic sample. Among these participants, 1,243 provided complete data across all three waves and were therefore retained for longitudinal stability stratification and subsequent subgroup analyses, corresponding to a three-wave retention rate of 89.1%. The remaining 152 participants (10.9%) had incomplete follow-up data and were not classified into the subsequent analyses.

Among participants with complete three-wave data, 953 (76.7%) were classified into the stability-dominant subgroup and 290 (23.3%) into the variability-dominant subgroup. Baseline demographic characteristics and baseline measurements for these two subgroups are summarized in Table 1. The two subgroups did not differ significantly in age, sex, grade, exercise self-efficacy, body image, or anxiety at baseline. A statistically significant between-group difference was observed for baseline physical activity distribution; however, the effect size was small (Cramér’s V = 0.07).

**Table 1:** Baseline demographic and measurement characteristics of the stability-dominant and variability-dominant subgroups.

Variable	Stability-dominant	Variability-dominant	Between-group comparison
*n* (%)	953 (76.7%)	290 (23.3%)	–
Age, years	13.01 ± 0.69	12.97 ± 0.70	SMD = 0.06; *p* = 0.403
Sex	–	–	Cramér’s V = 0.02; *p* = 0.576
Male	391 (41.0%)	125 (43.1%)	–
Female	562 (59.0%)	165 (56.9%)	–
Grade	–	–	Cramér’s V = 0.03; *p* = 0.397
Grade 7	467 (49.0%)	151 (52.1%)	—
Grade 8	486 (51.0%)	139 (47.9%)	—
Physical activity	–	–	Cramér’s V = 0.07; *p* = 0.039
Low	521 (54.7%)	156 (53.8%)	–
Moderate	127 (13.3%)	55 (19.0%)	–
High	305 (32.0%)	79 (27.2%)	–
Exercise self-efficacy	4.54 ± 2.00	4.48 ± 1.86	SMD = 0.03; *p* = 0.631
Body image	5.21 ± 1.98	5.20 ± 1.74	SMD = 0.01; *p* = 0.933
Anxiety	2.95 ± 0.55	2.98 ± 0.50	SMD = −0.05; *p* = 0.427

Values are presented as mean ± SD or *n* (%). Baseline measurements were obtained at T1. For continuous variables, between-group differences are summarized using standardized mean differences (SMD) and independent-samples *t*-test *p* values. For categorical variables, between-group differences are summarized using Cramér’s V and chi-square p values. SMD = standardized mean difference.

**Figure 1:**
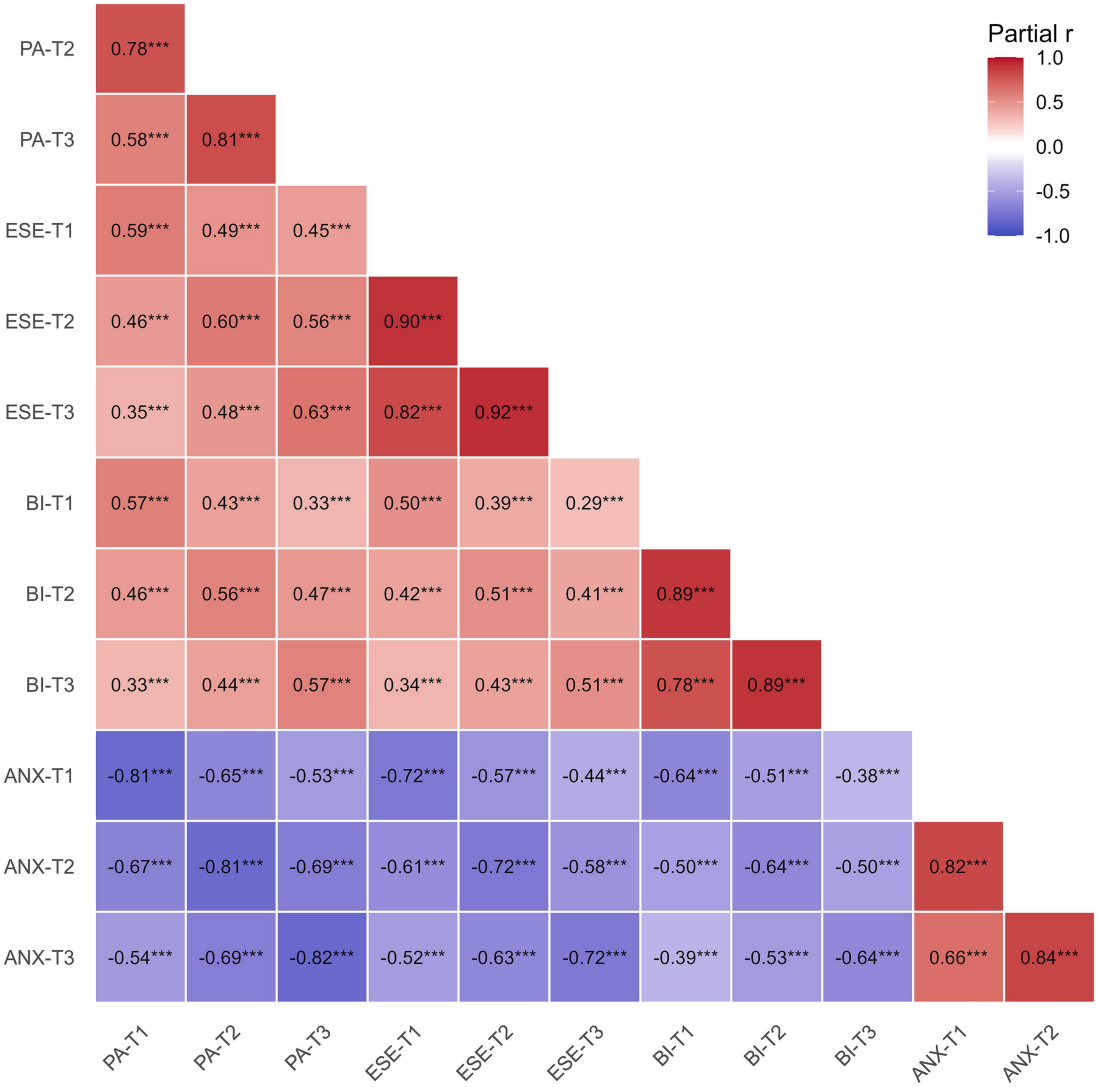
Partial correlation heatmap for the variability-dominant subgroup. Values represent partial correlations among physical activity (PA), exercise self-efficacy (ESE), body image (BI), and anxiety (ANX) across three waves, controlling for grade, sex, and age. *** denotes *p* < 0.001.

To further characterize the subgroup showing greater temporal variation, partial correlations among the four study domains across the three waves were examined within the variability-dominant subgroup while controlling for grade, sex, and age (Figure 1). The resulting correlation structure showed moderate-to-high within-domain cross-wave stability, with positive associations across physical activity, exercise self-efficacy, and body image over time. Anxiety was consistently and inversely associated with physical activity, exercise self-efficacy, and body image across waves. Overall, the variability-dominant subgroup retained sufficient longitudinal variation to support subsequent trajectory-based analyses.

### 4.2 Identification of anxiety developmental trajectories

Latent class trajectory models specifying one to five classes were estimated within the variability-dominant subgroup to examine heterogeneity in anxiety development across the three waves. As shown in Table 2, the 3-class solution showed substantially improved fit over the 2-class solution, with lower AIC and BIC values, while still maintaining strong classification quality (Entropy = 0.892; MeanPosteriorProb = 0.951). Although the 5-class solution yielded a slightly lower AIC than the 3-class solution, it produced a very small class (MinClassPct = 2.76) and weaker classification precision, indicating over-extraction. The 4-class solution was also inferior to the 3-class solution on both fit and classification indices. Accordingly, the 3-class solution was retained as the optimal model for subsequent analyses.

**Table 2:** Fit indices for anxiety trajectory models in the variability-dominant subgroup.

Classes	AIC	BIC	logLik	Entropy	MinClassPct	MeanPosteriorProb
1	589.2	611.2	−288.6	–	100.00	1.000
2	473.2	506.3	−227.6	0.926	20.69	0.979
3	392.6	436.7	−184.3	0.892	18.62	0.951
4	398.6	453.7	−184.3	0.620	19.66	0.711
5	388.1	454.1	−176.0	0.665	2.76	0.669

AIC = Akaike information criterion; BIC = Bayesian information criterion; logLik = log-likelihood. Entropy reflects classification precision, MinClassPct indicates the proportion of the smallest class in each solution, and MeanPosteriorProb represents the average posterior probability of assigned class membership. Entropy is not applicable to the 1-class solution.

**Figure 2:**
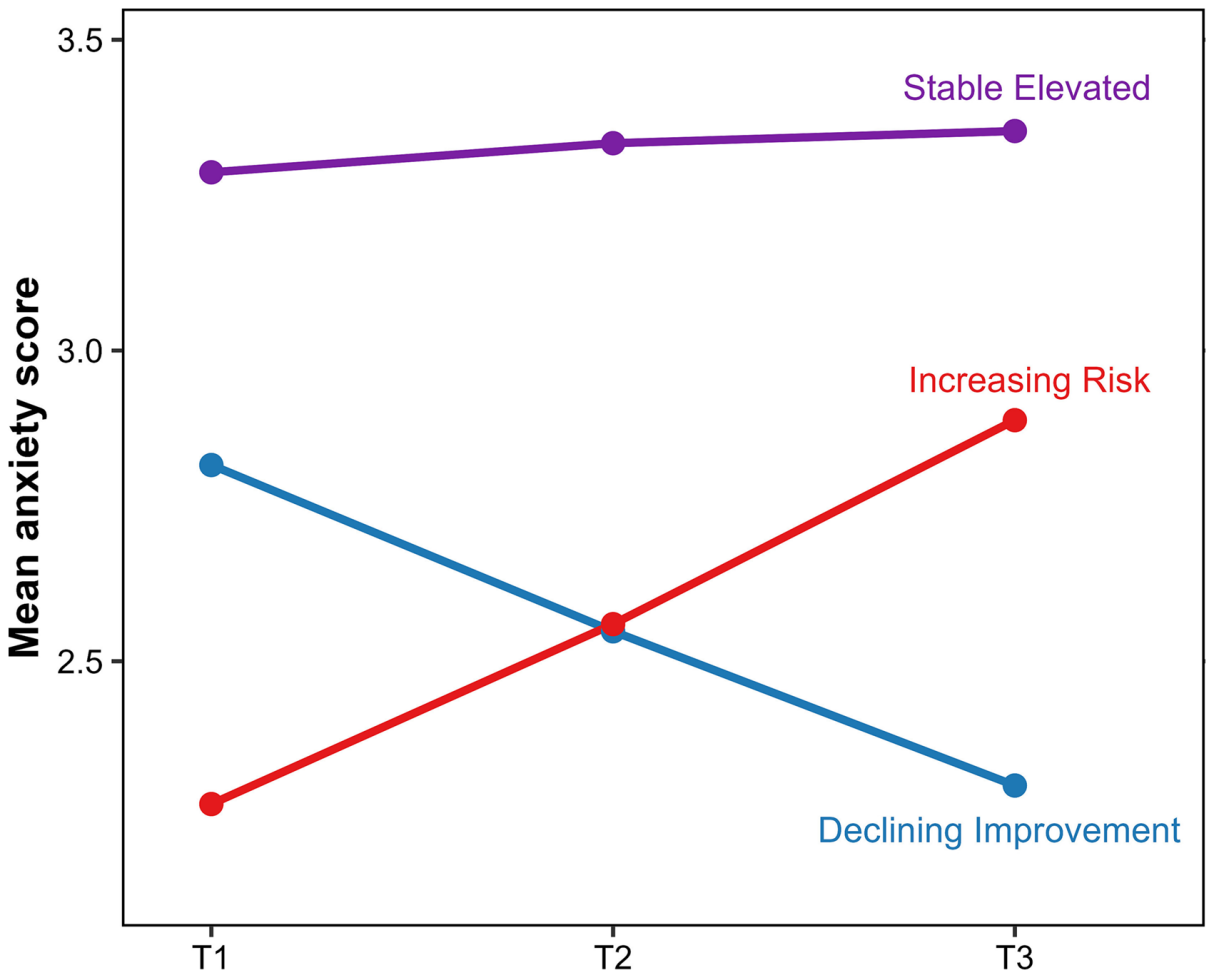
Retained 3-class anxiety trajectories in the variability-dominant subgroup.

The retained 3-class solution is illustrated in Figure 2 and summarized numerically in Table 3. The largest class, labeled Stable Elevated, showed persistently high anxiety across T1, T2, and T3. A second class, labeled Declining Improvement, showed a progressive decline in anxiety over time. The third class, labeled Increasing Risk, showed a gradual increase in anxiety across the study period. These findings indicate that adolescents in the variability-dominant subgroup did not follow a uniform developmental course, but instead separated into three distinct anxiety trajectories.

**Table 3:** Characteristics of the identified anxiety trajectory classes in the variability-dominant subgroup.

Trajectory class	*n*	%	ANX-T1	ANX-T2	ANX-T3
Stable elevated	174	60.00	3.29	3.33	3.35
Declining improvement	54	18.62	2.82	2.55	2.30
Increasing risk	62	21.38	2.27	2.56	2.89

Mean anxiety (ANX) values were derived from the 3-class solution in the variability-dominant subgroup. Class labels were assigned according to the observed trajectory patterns across T1–T3.

### 4.3 Multinomial predictors of anxiety trajectory membership

Baseline physical activity was positively associated with later exercise self-efficacy, such that adolescents with moderate and high physical activity reported higher exercise self-efficacy at T2 than those with low physical activity (Table 4). Demographic covariates were not significantly associated with exercise self-efficacy in this model. In the subsequent model, exercise self-efficacy was positively associated with body image at T3. Baseline physical activity also remained positively associated with later body image, and female participants reported more positive body image than male participants, whereas grade and age were not significant. Overall, higher baseline physical activity was associated with stronger later exercise self-efficacy and more positive later body image in the variability-dominant subgroup.

**Table 4:** Regression models for exercise self-efficacy and body image in the variability-dominant subgroup.

Predictor	Model 1: T2 exercise self-efficacy		Model 2: T3 body image	
	*β* (95% CI)	*p*	*β* (95% CI)	*p*
Moderate physical activity vs. low	1.18 (0.66, 1.70)	<0.001	0.81 (0.32, 1.30)	0.001
High physical activity vs. low	2.02 (1.56, 2.47)	<0.001	0.64 (0.16, 1.11)	0.008
Exercise self-efficacy (T2)	–	–	0.31 (0.21, 0.42)	<0.001
Grade 8 vs. Grade 7	0.00 (−0.54, 0.55)	0.986	0.06 (−0.44, 0.56)	0.812
Female vs. Male	−0.18 (−0.57, 0.21)	0.365	0.40 (0.04, 0.76)	0.028
Age	0.13 (−0.26, 0.52)	0.503	0.02 (−0.34, 0.37)	0.934

Low physical activity, Grade 7, and male were the reference categories.

Both the Declining Improvement and Increasing Risk classes differed from the Stable Elevated class (Table 5). Relative to the Stable Elevated class, membership in the Declining Improvement class was positively associated with baseline physical activity, stronger exercise self-efficacy, and more positive body image. By contrast, membership in the Increasing Risk class was positively associated with baseline physical activity and exercise self-efficacy, whereas body image did not significantly distinguish this class from the Stable Elevated class. Some demographic covariates additionally differentiated Declining Improvement from Stable Elevated, but not Increasing Risk. The odds ratios for baseline physical activity in the Increasing Risk contrast were notably large and should be interpreted cautiously, as they likely reflect a markedly uneven distribution of baseline physical activity across trajectory classes, with comparatively high baseline physical activity concentrated in the Increasing Risk group. Overall, exercise self-efficacy differentiated both nonreference trajectories from the Stable Elevated class, whereas body image was more specifically associated with the Declining Improvement trajectory.

**Table 5:** Multinomial logistic regression predicting anxiety trajectory membership in the variability-dominant subgroup.

Predictor	Declining improvement vs stable elevated		Increasing risk vs stable elevated	
	Odds ratio (95% CI)	*p*	Odds ratio (95% CI)	*p*
Moderate PA vs. Low	7.99 (2.42, 26.38)	<0.001	42.38 (4.68, 383.38)	<0.001
High PA vs. Low	21.22 (3.81, 118.25)	<0.001	1,756 (174, 17,692)	<0.001
Exercise self-efficacy (T2)	2.33 (1.64, 3.31)	<0.001	2.10 (1.45, 3.06)	<0.001
Body image (T3)	2.54 (1.69, 3.81)	<0.001	1.06 (0.69, 1.61)	0.802
Grade 8 vs. Grade 7	4.57 (1.15, 18.09)	0.031	1.12 (0.26, 4.83)	0.878
Female vs. Male	3.08 (1.05, 9.05)	0.040	2.05 (0.65, 6.45)	0.219
Age	0.51 (0.18, 1.44)	0.206	0.97 (0.32, 2.91)	0.954

Stable elevated was the reference trajectory class. Low physical activity (PA), Grade 7, and male were the reference categories.

### 4.4 Prospective indirect associations with anxiety trajectory membership

For the contrast between Declining Improvement and Stable Elevated (Table 6), baseline physical activity retained a significant direct association with membership in the Declining Improvement class. By contrast, the indirect pathways through exercise self-efficacy, through body image, and through the serial pathway linking exercise self-efficacy to body image were not supported, as the corresponding bootstrap confidence intervals all included zero. These findings indicate that, after adjustment for prior levels of exercise self-efficacy and body image, the association between baseline physical activity and membership in the Declining Improvement trajectory was primarily direct rather than indirectly transmitted through later exercise self-efficacy or body image. Variance inflation factors were low across predictors, with all values below 2.1, indicating no substantial multicollinearity.

**Table 6:** Bootstrapped indirect pathway estimates for declining improvement versus stable elevated in the variability-dominant subgroup.

PA contrast	Effect	Estimate	95% CI
Moderate PA vs. Low	Direct	1.955	0.779 to 3.972
	Indirect via exercise self-efficacy	0.137	−0.146 to 0.556
	Indirect via body image	0.130	−0.206 to 0.545
	Indirect via exercise self-efficacy → body image	0.026	−0.030 to 0.105
	Total indirect	0.293	−0.281 to 1.042
High PA vs. Low	Direct	3.362	2.411 to 6.440
	Indirect via exercise self-efficacy	0.147	−0.164 to 0.529
	Indirect via body image	0.116	−0.343 to 0.699
	Indirect via exercise self-efficacy → body image	0.027	−0.033 to 0.099
	Total indirect	0.290	−0.373 to 1.107

PA = physical activity.

A more complex pattern emerged for the contrast between Increasing Risk and Stable Elevated (Table 7). Baseline physical activity again showed a positive direct association with membership in the Increasing Risk class. At the same time, the indirect pathway through exercise self-efficacy was negative, and the total indirect effect was likewise negative, whereas the indirect pathway through body image and the serial exercise self-efficacy-to-body-image pathway were not supported. The negative indirect effect through exercise self-efficacy for the Increasing Risk contrast is not directly comparable to the positive multinomial odds ratio reported in Table 5. The indirect pathway estimates were derived from separate contrast-specific binary models with prior mediator adjustment and are reported on the coefficient scale rather than the odds-ratio scale. This configuration suggests an inconsistent indirect pattern, in which the pathway through later exercise self-efficacy may partially offset the direct association of baseline physical activity with Increasing Risk class membership. Interpretation of this contrast nevertheless requires caution. Baseline physical activity was distributed very unevenly across trajectory classes, with high physical activity concentrated particularly strongly in the Increasing Risk class. Multicollinearity was unlikely to account for these findings, as variance inflation factors again remained low throughout, with all values below 2.1.

**Table 7:** Bootstrapped indirect pathway estimates for increasing risk versus stable elevated in the variability-dominant subgroup.

PA contrast	Effect	Estimate	95% CI
Moderate PA vs. Low	Direct	3.650	2.105 to 22.494
	Indirect via exercise self-efficacy	−0.365	−0.967 to −0.150
	Indirect via body image	−0.332	−1.286 to 0.221
	Indirect via exercise self-efficacy → body image	−0.006	−0.040 to 0.007
	Total indirect	−0.703	−1.888 to −0.290
High PA vs. Low	Direct	7.387	6.238 to 30.341
	Indirect via exercise self-efficacy	−0.510	−1.283 to −0.218
	Indirect via body image	−0.546	−2.038 to 0.337
	Indirect via exercise self-efficacy → body image	−0.009	−0.058 to 0.009
	Total indirect	−1.064	−2.754 to −0.405

PA = physical activity.

These analyses did not support a consistent serial indirect pathway. Instead, the pattern was more consistent with prospective indirect associations that differed across trajectory contrasts. For the Declining Improvement contrast, baseline physical activity was linked to trajectory membership chiefly through a direct association. For the Increasing Risk contrast, exercise self-efficacy emerged as a significant countervailing indirect pathway, whereas body image did not function as a reliable indirect mechanism.

Taken together, Hypothesis 4 was supported, as exercise self-efficacy positively predicted subsequent body image. Hypothesis 1 received partial support, insofar as baseline physical activity differentiated anxiety trajectory membership, but not exclusively in the direction of lower-risk trajectories. Hypotheses 2, 3, and 5 were not supported in their originally proposed mediation form, as the indirect pathways through exercise self-efficacy, through body image, and through the serial exercise self-efficacy-to-body-image pathway were not consistently supported across trajectory contrasts.

## 5 Discussion

This study suggests that the role of physical activity in adolescent anxiety is better understood developmentally than cross-sectionally. Within the variability-dominant subgroup, anxiety did not evolve along a single common pathway across one academic year. Instead, adolescents separated into stable elevated, declining-improvement, and increasing-risk trajectories. This distinction matters because it shifts the question from whether physical activity is associated with lower anxiety in general to whether it is associated with different forms of anxiety development over time. Recent longitudinal studies likewise indicate that adolescent internalizing symptoms are structured by heterogeneous trajectories rather than a single average course (34–36). Our results extend that work by placing physical activity within a person-centered developmental framework, where its relevance lies in differential positioning across anxiety pathways rather than in mean symptom differences alone.

The physical activity findings also resist a simple protective-factor reading. A broad literature already shows that physically active adolescents tend to report better mental health, and newer work suggests that these associations may be nonlinear rather than uniformly linear (37–39). The current findings are compatible with that literature, but they add an important qualification. Physical activity differentiated trajectory membership, yet the pattern was not reducible to a simple “more is always better” account. Conceptually, physical activity may be better conceived as a developmental resource whose psychological value depends on how it is embedded in adolescents’ broader self-regulatory and social contexts. This is a more precise claim than the familiar view of physical activity as a uniformly protective factor.

The mechanism pattern refines this account further. The clearest pattern was not the full serial pathway originally hypothesized, but an asymmetry between competence-related and evaluative processes. Baseline physical activity positively predicted later exercise self-efficacy, and exercise self-efficacy positively predicted later body image. This is broadly consistent with newer work showing that physical activity may support adolescent adjustment through self-related processes such as efficacy, self-esteem, resilience, and stress management (21, 40, 41). The added value of the present study is that these processes were examined against longitudinal anxiety pathways rather than single-wave well-being indicators. Within this framework, exercise self-efficacy appeared to operate as the more general and theoretically stable correlate, whereas body image played a narrower role.

That distinction is substantively important. Body image did not emerge as an equally robust pathway across both trajectory contrasts, and the serial indirect pathway was not consistently supported. This result helps refine the theory. The pathway linking physical activity to anxiety development may be anchored more strongly in perceived capability than in appearance-related self-evaluation. Body image still appears relevant, particularly for more adaptive movement away from persistently elevated anxiety, but it did not function here as a universal explanatory bridge. Recent longitudinal work on body image supports this more bounded reading by showing that body-related self-evaluation is patterned, gradual, and context-sensitive rather than uniformly unstable across adolescence (42, 43). Our results therefore reposition body image from a presumed central mechanism to a more conditional component of the developmental process.

A second contribution lies in the coexistence of stability and heterogeneity. Most adolescents with complete three-wave data were classified into the stability-dominant subgroup, whereas trajectory analyses were conducted in the smaller subgroup that retained greater variation. This should not be treated only as a methodological complication. It suggests that over a one-year interval in a relatively homogeneous middle-school setting, physical activity, exercise self-efficacy, body image, and anxiety may function for many adolescents more like relatively stable psychosocial profiles than rapidly fluctuating states. Newer longitudinal evidence similarly shows that stable groups are common in adolescent internalizing research even when smaller subgroups still diverge meaningfully, and that body-image change is often gradual rather than abrupt (34, 42–44). Our results therefore suggest that heterogeneity should not be conflated with pervasive malleability. Developmental pathways can diverge, but they do so against a substantial background of continuity.

The study does more than show that some adolescents improve while others worsen. It shows that anxiety development may be structured simultaneously by continuity at the broader sample level and by differentiation within a smaller, more variable subgroup. That dual pattern matters for theory because it suggests that models of adolescent anxiety should account for both relatively stable risk profiles and subgroup-specific developmental drift. Within that framework, physical activity may matter less because it eliminates baseline vulnerability and more because it is associated with how that vulnerability is expressed across time. This is a subtler but stronger developmental claim than a general symptom-reduction narrative.

Several limitations define the scope of these conclusions. First, the trajectory and pathway analyses were restricted to the variability-dominant subgroup rather than the full retained sample. Second, the follow-up covered only one academic year, which limits inference about longer developmental reorganization. Third, although temporal ordering was imposed, the indirect effects are more appropriately interpreted as prospective associations than as strong causal mediation. These constraints also point directly to the next step. Recent work shows that wearables and smartphone-based ecological momentary assessment are increasingly feasible in child and adolescent mental-health research and can capture activity and emotional fluctuation with much greater temporal precision than wave-separated self-report (45, 46). For this topic, such designs could help determine whether the large stability-dominant subgroup reflects true psychosocial continuity, limited within-person sensitivity of questionnaire methods, or both.

## 6 Practical implications

The practical implications are clearest when physical activity is viewed as a competence-building context rather than as a generic health behavior. Reduced to the claim that exercise benefits mental health, the present findings add little. A more useful interpretation is that school-based physical activity may matter when it repeatedly gives adolescents opportunities to experience progress, successful engagement, and manageable challenge. In that sense, the value of physical education may lie not only in movement volume, but in how activity settings organize mastery experiences. This view is consistent with broader school-based prevention literature showing that mental-health effects are typically modest, context-sensitive, and dependent on program quality rather than mere exposure (47).

The mechanism pattern also argues against heavily appearance-centered intervention logic. Because exercise self-efficacy was more consistently implicated than body image, programs that focus primarily on body shape, weight, or appearance comparison may miss the more central process suggested by the present results. A closer fit to the evidence would be activity environments that emphasize progressive skill development, attainable goals, encouragement, and feedback tied to capability rather than appearance. This may be especially relevant in adolescence, when evaluative comparison can amplify vulnerability instead of reducing it. The practical message, then, is not simply to increase physical activity opportunities, but to design them in ways that strengthen adolescents’ sense of competence and sustained engagement.

## 7 Conclusion

Physical activity contributes more clearly to understanding adolescent anxiety when anxiety is treated as a heterogeneous developmental process rather than as a single average outcome. Within the variability-dominant subgroup, anxiety followed three distinct pathways, and baseline physical activity was associated with differential trajectory membership across those pathways. More importantly, the current findings indicate that the explanatory value of physical activity lies less in a broad claim of universal benefit than in a more specific developmental account: physical activity appears to be linked to anxiety pathways more consistently through competence-related processes than through appearance-related ones, and these associations unfold against a broader background of psychosocial stability. Taken together, our results refine the literature by showing that adolescent anxiety development is shaped not only by risk level, but also by how stability, heterogeneity, and self-appraisal intersect over time.

## Data Availability

The raw data supporting the findings of this study are available from the corresponding author (Y-XF) upon reasonable request.

## References

[1] Beesdo-Baum K Knappe S. Developmental epidemiology of anxiety disorders. Child Adolesc Psychiatr Clin North Am. (2012) 21:457–78. doi: 10.1016/j.chc.2012.05.001, 22800989

[2] Merikangas KR He J-p Burstein M Swanson SA Avenevoli S Cui L . Lifetime prevalence of mental disorders in U.S. Adolescents: results from the national comorbidity survey replication–adolescent supplement (NCS-A). J Am Acad Child Adolesc Psychiatry. (2010) 49:980–9. doi: 10.1016/j.jaac.2010.05.017, 20855043 PMC2946114

[3] Cummings CM Caporino NE Kendall PC. Comorbidity of anxiety and depression in children and adolescents: 20 years after. Psychol Bull. (2014) 140:816–45. doi: 10.1037/a0034733, 24219155 PMC4006306

[4] Morales-Muñoz I Mallikarjun PK Chandan JS Thayakaran R Upthegrove R Marwaha S. Impact of anxiety and depression across childhood and adolescence on adverse outcomes in young adulthood: a UK birth cohort study. Br J Psychiatry. (2023) 222:212–20. doi: 10.1192/bjp.2023.23, 36919351 PMC10895507

[5] Gallagher C Pirkis J Lambert KA Perret JL Ali GB Lodge CJ . Life course BMI trajectories from childhood to mid-adulthood are differentially associated with anxiety and depression outcomes in middle age. Int J Obes. (2023) 47:661–8. doi: 10.1038/s41366-023-01312-6, 37161067 PMC10359183

[6] Singh B Bennett H Miatke A Dumuid D Curtis R Ferguson T . Systematic umbrella review and meta-meta-analysis: effectiveness of physical activity in improving depression and anxiety in children and adolescents. J Am Acad Child Adolesc Psychiatry. (2026) 65:171–86. doi: 10.1016/j.jaac.2025.04.007, 40239946

[7] Li X Chen W Zhang Z Li Z Fan X Ma T . Association between physical activity and risk of anxiety: a dose-response meta-analysis of 11 international cohorts. EClinicalMedicine. (2025) 84:103285. doi: 10.1016/j.eclinm.2025.103285, 40534999 PMC12173747

[8] White RL Vella S Biddle S Sutcliffe J Guagliano JM Uddin R . Physical activity and mental health: a systematic review and best-evidence synthesis of mediation and moderation studies. Int J Behav Nutr Phys Act. (2024) 21:134. doi: 10.1186/s12966-024-01676-6, 39609855 PMC11603721

[9] Bandura A Self-Efficacy. The Exercise of Control, vol. ix New York, NY, US: W H Freeman/Times Books/ Henry Holt & Co (1997). p. 604.

[10] Liu L Yisongwake A Hao Y Lyu Z Zhao Z Wang Z . The association between physical activity and positive affect in adolescents: the chain mediating role of psychological resilience and regulatory emotional self-efficacy. Psychol Health Med. (2024) 29:1807–19. doi: 10.1080/13548506.2024.2411635, 39377294

[11] Wu J Shao Y Hu J Zhao X. The impact of physical exercise on adolescent social anxiety: the serial mediating effects of sports self-efficacy and expressive suppression. BMC Sports Sci Med Rehabil. (2025) 17:57. doi: 10.1186/s13102-025-01107-4, 40121514 PMC11929206

[12] Vannucci A Ohannessian CM. Body image dissatisfaction and anxiety trajectories during adolescence. J Clin Child Adolesc Psychol. (2018) 47:785–95. doi: 10.1080/15374416.2017.1390755, 29087230 PMC6072626

[13] Yurdagül C Kircaburun K Emirtekin E Wang P Griffiths MD. Psychopathological consequences related to problematic Instagram use among adolescents: the mediating role of body image dissatisfaction and moderating role of gender. Int J Ment Health Addict. (2021) 19:1385–97. doi: 10.1007/s11469-019-00071-8

[14] Thompson JK Heinberg LJ Altabe M Tantleff-Dunn S. Exacting Beauty: Theory, Assessment, and Treatment of Body Image Disturbance, vol. xii Washington, DC, US: American Psychological Association (1999). p. 396.

[15] Cicchetti D Rogosch FA. A developmental psychopathology perspective on adolescence. J Consult Clin Psychol. (2002) 70:6–20. doi: 10.1037/0022-006X.70.1.6, 11860057

[16] Zhu X Huebner ES Tian L. A person-centered longitudinal analysis of adolescents’ loneliness and social anxiety: clusters, predictors, and outcomes. School Psychol. (2019) 34:576–89. doi: 10.1037/spq0000328 31246063

[17] Yuan Y Yang J Huang W Hu C Zhang W Chen B. Relationships among anxiety, psychological resilience, and physical activity in university students: variable-centred and person-centred perspectives. Front Psychol. (2025) 16:1694344. doi: 10.3389/fpsyg.2025.1694344, 41356033 PMC12679388

[18] Merikangas KR Zhang H Avenevoli S Acharyya S Neuenschwander M Angst J. Longitudinal trajectories of depression and anxiety in a prospective community study: the Zurich cohort study. Arch Gen Psychiatry. (2003) 60:993–1000. doi: 10.1001/archpsyc.60.9.993, 14557144

[19] Krygsman A Vaillancourt T. Elevated social anxiety symptoms across childhood and adolescence predict adult mental disorders and cannabis use. Compr Psychiatry. (2022) 115:152302. doi: 10.1016/j.comppsych.2022.152302, 35245889

[20] Mendes RA Loxton NJ Browning NG Lawrence RK. The effect of psychological interventions on statistics anxiety, statistics self-efficacy, and attitudes toward statistics in university students: a systematic review. Educ Psychol Rev. (2025) 37:3. doi: 10.1007/s10648-024-09979-7

[21] Zhang G Feng W Zhao L Zhao X Li T. The association between physical activity, self-efficacy, stress self-management and mental health among adolescents. Sci Rep. (2024) 14:5488. doi: 10.1038/s41598-024-56149-4, 38448518 PMC10917799

[22] Mu F-Z Liu J Lou H Zhu W-D Wang Z-C Li B. How breaking a sweat affects mood: the mediating role of self-efficacy between physical exercise and emotion regulation ability. PLoS One. (2024) 19:e0303694. doi: 10.1371/journal.pone.0303694, 38870188 PMC11175485

[23] Taherifard M Ghahremani L Gheibi Z Saeidmanesh M Vahab M. Comparison of body image dissatisfaction among adolescent boys who do and do not stutter and investigating the relationship between body image dissatisfaction, anxiety and self-assessed stuttering severity. BMC Psychol. (2025) 13:893. doi: 10.1186/s40359-025-03266-y, 40790501 PMC12341262

[24] Thorup L Zulfikari M Sørensen CLB Biering K. Body image and depressive symptoms in Danish adolescents: a cross-sectional national study. J Affect Disord. (2024) 365:65–72. doi: 10.1016/j.jad.2024.08.016, 39153549

[25] Mills JS Minister C Samson L. Enriching sociocultural perspectives on the effects of idealized body norms: integrating shame, positive body image, and self-compassion. Front Psychol. (2022) 13:983534. doi: 10.3389/fpsyg.2022.983534, 36506975 PMC9732395

[26] Frederick DA Tylka TL Rodgers RF Pennesi J-L Convertino L Parent MC . Pathways from sociocultural and objectification constructs to body satisfaction among women: the U.S. body project I. Body Image. (2022) 41:195–208. doi: 10.1016/j.bodyim.2022.02.001, 35299008 PMC9764838

[27] Spurr JM Stopa L. Self-focused attention in social phobia and social anxiety. Clin Psychol Rev. (2002) 22:947–75. doi: 10.1016/S0272-7358(02)00107-1 12238248

[28] Zhu J Jiang Z Li Y Cai Y Chen J. The mechanism of body appreciation influencing social anxiety in college students: a moderated mediation model. Acta Psychol. (2025) 255:104884. doi: 10.1016/j.actpsy.2025.104884, 40068476

[29] Laing D. 高校学生应激水平及其与体育锻炼的关系 [stress level of college students and its relationship with physical exercise]. Chin Ment Health J. (1994) 1:5–6.

[30] Lee L-L Perng S-J Ho C-C Hsu H-M Lau S-C Arthur A. A preliminary reliability and validity study of the Chinese version of the self-efficacy for exercise scale for older adults. Int J Nurs Stud. (2009) 46:230–8. doi: 10.1016/j.ijnurstu.2008.09.003, 18950769

[31] Cash TF Fleming EC Alindogan J Steadman L Whitehead A. Beyond body image as a trait: the development and validation of the body image states scale. Eat Disord. (2002) 10:103–13. doi: 10.1080/10640260290081678, 16864251

[32] Zung WWK. A rating instrument for anxiety disorders. Psychosomatics. (1971) 12:371–9. doi: 10.1016/S0033-3182(71)71479-0 5172928

[33] Proust-Lima C Philipps V Liquet B. Estimation of extended mixed models using latent classes and latent processes: the R package LCMM. J Stat Softw. (2017) 78:1–56. doi: 10.18637/jss.v078.i02

[34] Cetinkaya D DeCaro SA Turnamian MR Poon JA Kleiman EM Liu RT. A multi-method assessment of emotional processes predicting longitudinal anxiety symptom trajectories in an adolescent clinical sample. J Mood Anxiety Disord. (2024) 7:100071. doi: 10.1016/j.xjmad.2024.100071 39421464 PMC11485166

[35] Li M Huang Z Ding J Wu H Liang S. Longitudinal trajectories of depressive symptoms in adolescent students. BMC Psychol. (2025) 14:90. doi: 10.1186/s40359-025-03874-8, 41408363 PMC12821241

[36] Liu X Zhang Y Gao W Cao X. Developmental trajectories of depression, anxiety, and stress among college students: a piecewise growth mixture model analysis. Humanit Soc Sci Commun. (2023) 10:736. doi: 10.1057/s41599-023-02252-2

[37] Zhang Y Wang Z Zhang R Wang Y Yang J Wang F . Non-linear dose-response association between physical activity and mental health in adolescents: a prospective cohort study based on search. Int J Behav Nutr Phys Act. (2025) 22:146. doi: 10.1186/s12966-025-01848-y, 41267100 PMC12632063

[38] Zhou H Jiang F Liu H Wu Y Tang Y-l. Dose-dependent association between physical activity and mental health, and mitigation effects on risk behaviors. iScience. (2025) 28:111866. doi: 10.1016/j.isci.2025.111866, 39991549 PMC11847119

[39] Chen Y Song J Zhao G Fu J. The impact of physical exercise on adolescent psychological resilience: an empirical analysis based on deep neural networks. Front Psychol. (2026) 16:1709478. doi: 10.3389/fpsyg.2025.1709478, 41567441 PMC12816237

[40] Laurier C Pascuzzo K Jubinville V Lemieux A. Physical activity and its benefits on adolescents' mental health through self-esteem. Front Child Adolesc Psychiatry. (2024) 3:1503920. doi: 10.3389/frcha.2024.1503920 39816571 PMC11732102

[41] Yu X Wu M Wang J Yang Y Qiu Q Yuan Y . The relationship between physical activity and social adjustment in adolescents: the longitudinal mediating role of emotional resilience. BMC Public Health. (2026) 26:734. doi: 10.1186/s12889-026-26271-1 41618252 PMC12933968

[42] Gualdi-Russo E Masotti S Rinaldo N De Luca F Toselli S Mazzoni G . A longitudinal study on body image perception and size among Italian early adolescents: changes over time and discrepancies between genders. Nutrients. (2024) 16:3439. doi: 10.3390/nu16203439, 39458435 PMC11510257

[43] Lacroix E Smith AJ Husain IA Orth U von Ranson KM. Normative body image development: a longitudinal meta-analysis of mean-level change. Body Image. (2023) 45:238–64. doi: 10.1016/j.bodyim.2023.03.003, 36965235

[44] van Loon AWG Creemers HE Vogelaar S Saab N Miers AC Westenberg PM . Trajectories of adolescent perceived stress and symptoms of depression and anxiety during the Covid-19 pandemic. Sci Rep. (2022) 12:15957. doi: 10.1038/s41598-022-20344-y 36153394 PMC9509354

[45] Bergwerff CE Buisman RSM Nibbering N Noordermeer SDS. Using wearables in mental health care for children and adolescents: a scoping review. Res Child Adolesc Psychopathol. (2026) 54:16. doi: 10.1007/s10802-025-01408-9, 41588150 PMC12835090

[46] Lutz L Veltmann P Meisterhans R Giurgiu M. Associations between wearables vital parameters and self-perceived mood—an ecological momentary assessment study among healthy adolescents. Front Psychol. (2025) 16:1623886. doi: 10.3389/fpsyg.2025.1623886, 41509153 PMC12777082

[47] Werner-Seidler A Spanos S Calear AL Perry Y Torok M O'Dea B . School-based depression and anxiety prevention programs: an updated systematic review and meta-analysis. Clin Psychol Rev. (2021) 89:102079. doi: 10.1016/j.cpr.2021.102079, 34571372

